# Unsupervised Flow Cytometry Analysis Allows for an Accurate Identification of Minimal Residual Disease Assessment in Acute Myeloid Leukemia

**DOI:** 10.3390/cancers13040629

**Published:** 2021-02-05

**Authors:** Jean Philippe Vial, Nicolas Lechevalier, Francis Lacombe, Pierre-Yves Dumas, Audrey Bidet, Thibaut Leguay, François Vergez, Arnaud Pigneux, Marie C. Béné

**Affiliations:** 1 Hematology Biology, Flow Cytometry, Bordeaux University Hospital, 33600 Pessac, France; jean-philippe.vial@chu-bordeaux.fr (J.P.V.); nicolas.lechevalier@chu-bordeaux.fr (N.L.); francis.lacombe@chu-bordeaux.fr (F.L.); 2 Service d’Hématologie Clinique et de Thérapie Cellulaire, Bordeaux University Hospital, 33600 Pessac, France; pierre-yves.dumas@chu-bordeaux.fr (P.-Y.D.); thibaut.leguay@chu-bordeaux.fr (T.L.); arnaud.pigneux@chu-bordeaux.fr (A.P.); 3 Hematology Biology, Molecular Hematology, Bordeaux University Hospital, 33600 Pessac, France; audrey.bidet@chu-bordeaux.fr; 4 Hematology Biology, IUCT Oncopôle, Toulouse University Hospital, 31000 Toulouse, France; vergez.Francois@iuct-oncopole.fr; 5 Hematology Biology, Nantes University Hospital, 44000 Nantes, France

**Keywords:** acute myeloid leukemia, molecular markers, multiparameter flow cytometry, minimal/measurable residual disease, unsupervised analysis, FlowSOM

## Abstract

**Simple Summary:**

In acute myeloid leukemia (AML), minimal/measurable residual disease (MRD) can be assessed based on molecular markers or immunophenotypic features evaluated at diagnosis, through multiparameter flow cytometry (MFC) for the latter. New artificial intelligence tools allow to perform unsupervised analysis of MFC data. The Flow-Self-Organizing-Maps (FlowSOM) tool was used here to concomitantly compare MFC features of normal bone marrow together with diagnosis and follow-up bone marrow samples from 40 AML patients for the evaluation of MRD. MFC results were compared to molecular MRD, showing high concordance. This opens the road for a new easy and objective way of assessing MRD even in AML patients without molecular markers.

**Abstract:**

The assessment of minimal residual disease (MRD) is increasingly considered to monitor response to therapy in hematological malignancies. In acute myeloblastic leukemia (AML), molecular MRD (mMRD) is possible for about half the patients while multiparameter flow cytometry (MFC) is more broadly available. However, MFC analysis strategies are highly operator-dependent. Recently, new tools have been designed for unsupervised MFC analysis, segregating cell-clusters with the same immunophenotypic characteristics. Here, the Flow-Self-Organizing-Maps (FlowSOM) tool was applied to assess MFC-MRD in 96 bone marrow (BM) follow-up (FU) time-points from 40 AML patients with available mMRD. A reference FlowSOM display was built from 19 healthy/normal BM samples (NBM), then simultaneously compared to the patient’s diagnosis and FU samples at each time-point. MRD clusters were characterized individually in terms of cell numbers and immunophenotype. This strategy disclosed subclones with varying immunophenotype within single diagnosis and FU samples including populations absent from NBM. Detectable MRD was as low as 0.09% in MFC and 0.051% for mMRD. The concordance between mMRD and MFC-MRD was 80.2%. MFC yielded 85% specificity and 69% sensitivity compared to mMRD. Unsupervised MFC is shown here to allow for an easy and robust assessment of MRD, applicable also to AML patients without molecular markers.

## 1. Introduction

The prognosis of acute myeloblastic leukemia (AML) has considerably improved in the past few years through progress in therapeutic schedules, emergence of new therapies and a better definition of risk-groups. AMLs are very heterogeneous hematological malignancies, but three prognostic risk groups have been defined by the European LeukemiaNet (ELN) in 2017, respectively with favorable, intermediate and poor outcome, taking into account cytogenetic and molecular criteria [1]. However, although about 80% of patients reach complete remission (CR) after one cycle of chemotherapy, nearly 50% of them will relapse [2,3]. The current definition of CR is still based on morphological examination of a bone marrow (BM) smear disclosing less than 5% blasts [4]. It is however largely admitted that this threshold and this technique do not allow to detect small numbers of persisting leukemic cells, called ”Measurable Residual Disease” (MRD) and formerly “Minimal Residual Disease”.

MRD detection has become one of the major challenges to adapt therapy and prognosis. Several methods have been developed, allowing to reach very low detection thresholds: reverse transcriptase-quantitative PCR (RT-qPCR), multiparameter flow cytometry (MFC), next-generation sequencing (NGS) and, more recently, digital droplet PCR (ddPCR) [5]. These various techniques are complementary in the evaluation of MRD and ELN recommends to combine them [6]. RT-qPCR allows to track the decrease of PML-RARA transcripts, NPM1 mutations, as well as RUNX1-RUNX1T1 and CBFB-MYH11 anomalies. This method is very specific and sensitive but only relates to subgroups of AML, representing about 50% of the patients [7]. NGS requires great expertise in the interpretation of bioinformatic data and the delays and costs of such analyses remain elevated. However, recent publications [8,9], using NGS panels, report on the presence of at least one mutation in more than 80% of AML patients.

MFC has been largely demonstrated to be a mandatory diagnostic tool as well as being quite performing for the follow-up (FU) of any type of AML, independently of molecular anomalies. There is a consensus about the fact that “different-from-normal (DfN)” and “Leukemia-associated immunophenotype (LAIP)” approaches are congruent [6]. The “DfN” approach, by using a fixed panel of antibodies, describes immunophenotypic aberrations by comparison to physiological maturation profiles defined on normal BM (NBM) samples and indeed covers the most frequent LAIP. The LAIP concept defines immunophenotypic aberrations at diagnosis and tracks them during FU. The latter can be underexpression of a given marker, overexpression, asynchronous expression or aberrant expression of a marker usually expressed on another lineage [6]. Unfortunately, exhaustive identification of LAIP could require a large number of conjugated-antibody combinations, possibly dramatically increasing the cost of the method and complicating the comparison with enough NBM counterparts. Moreover, the LAIP approach can be limited by immunophenotypic modulation on blast cells or emergence of a sub-clone with potential for relapse, increasing the risk of falsely negative MRD [10]. Of note, absence of systematic comparison with NBM may also lead to the possibility of false positive MRD.

Thanks to the huge progress in MFC in the past 15 years, increasing numbers of parameters can now be analyzed simultaneously on small samples. However, information increases in parallel and makes classical bi-parametric interpretation more and more complex. Gating of the blastic population of interest remains highly subjective and operator-dependent as well as the potential observation of subclones. Thus, the major drawbacks of universal MFC analysis of MRD are the increasing number of parameters to take into account and lack of harmonization between panels. In 2018, the ELN produced recommendations, and particularly a combined approach integrating “DfN” and “LAIP” [6]. Simultaneously, the ELN mentioned that the use of new software, based on non-supervised analysis, should be considered in order to reduce interpretation subjectivity. Initially used for mass-cytometry data, such new algorithms are based on a reduction of the number of dimensions (principal component analysis (PCA), t-distributed stochastic neighbor embedding (t-SNE), visualizing data using t-SNE (viSNE)) or on clustering methods (Spanning-tree Progression Analysis of Density-normalized Events (SPADE), Citrus, PhenoGraph, Flow-Self Organizing Maps (FlowSOM)) [11,12,13,14,15,16,17,18,19]. Although most of them can be adapted to treat classical MFC data, these different solutions are not well adapted to MRD analyses: the number of events to process needs to be quite high to obtain a good sensitivity and software-based analysis time can be very long, up to several hours and thus incompatible with daily routine. Among these methods, the FlowSOM unsupervised solution, included in the open-access R software (Bioconductor), appears to be the best adapted for routine work and especially for MRD analysis in AML. It combines a short analysis time, a high sensitivity [11] and complies to ELN recommendations by combining “DfN” and “LAIP” approaches.

We have previously explored and published [13,15] how FlowSOM can be integrated with such classical analysis software as Kaluza^®^. In this work, we aimed at appreciating the performance of the FlowSOM solution together with Kaluza^®^ in real-life for MRD assessment. This was achieved by comparison to molecular analyses in the evaluation of MRD in 40 AML patients who received a classical intensive chemotherapy treatment. A total number of 96 follow-up (FU) points was analyzed, including FU1 (MRD1 at the end of induction) for all 40 patients.

## 2. Materials and Methods

### 2.1. Patients and Samples

From the laboratory database of Bordeaux University Hospital (Bordeaux, France), diagnosis data were selected from untreated AML (excluding promyelocytic AML) patients who had been tested between April 2015 and March 2020 and carried a molecular marker allowing for molecular FU. Diagnosis had been performed by morphologic examination according to the WHO classification [20]. All patients were treated by an intensive classical chemotherapy regimen based on a 3 + 7 backbone combining 3 days of anthracyclin and 7 days of cytarabine [21]. Among these patients, only those who benefited from FU by both molecular methods and MFC, at least for the post-induction time-point, were retained. Whenever further matched FU points were available, they were also collected and analyzed. Overall, 40 patients were included (24 men and 16 women, mean age 54 years old, Table 1 and Table S1), with 96 MRD points of FU assayed between 1- and 28-months post diagnosis. Written informed consent was obtained from all patients in accordance with the Declaration of Helsinki, allowing the collection of bioclinical data in the anonymized Bordeaux DATAML registry (authorization n°915285). MFC analysis at diagnosis and all time points was performed on whole BM samples collected on EDTA-K in a stain-lysis-no wash fashion. Two 10 color antibody combinations were used, according to recommendations of the GEIL (Groupe d’Etude Immunologique des Leucémies) as published before [22,23]. A common backbone of CD33, CD34 and CD45 was present in both tubes. One combination (tube 1) then associated CD65, CD14, CD13, CD117, CD7, CD11b and CD16 and the other one (tube 2) comprised CD64, CD10, CD4, CD123, CD56, CD19 and CD38. All antibodies were from Beckman-Coulter (Miami, FL, USA) and samples were processed on Navios instruments (Beckman-Coulter) harmonized according to Harmonemia recommendations [24].

In parallel, as reported previously [13], 19 NBM samples had been obtained and analyzed in MFC on the same instruments with the same antibody combinations to construct reference unsupervised immunophenotypic patterns for both tubes.

### 2.2. Molecular Assays

Molecular anomalies used for FU involved 29 patients with *NPM1* variant (24 type A, 3 type B, 2 type D), 6 with *RUNX1-RUNX1T1*, 4 with *CBFB-MYH11* and 1 with *BCR-ABL1* (Table 1).

RNAs were purified from BM mononuclear cells using Trizol reagent (Invitrogen-Thermo Fischer, Carlsbad, CA, USA). Reverse transcription was performed using the Transcriptor First Strand cDNA Synthesis Kit (Lifetechnologies-Thermo Fischer, Carlsbad, CA, USA). Molecular residual disease was assessed for *NPM1* variants, *RUNX1-RUNX1T1*, *CBFB-MYH11* and *BCR-ABL1* fusion transcripts as previously described [25,26]. The assessment of transcript levels was performed on a Light Cycler 480 (Roche LifeScience, Penzberg, Germany) with a specific RQ-PCR assay for each transcript. MRD levels were reported as the normalized values of fusion transcripts or *NPM1mut* copy number/*ABL* copy number × 100 (%). The quantitative detection limit of the assays was 13 copies for *NPM1* mutations and 3 copies for fusion transcripts. The achievement of MRD levels below these thresholds was defined as a negative MRD.

### 2.3. FlowSOM Process

**Figure 1:**
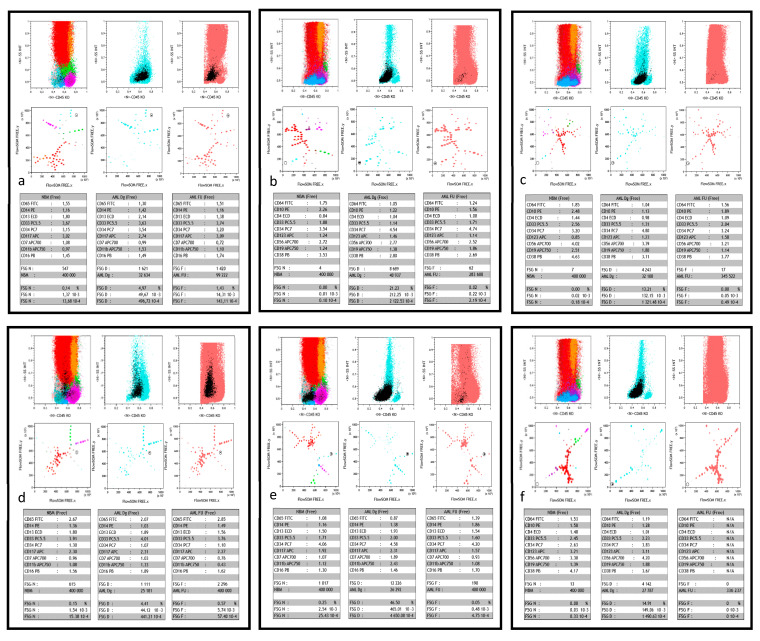
Typical FlowSOM/Kaluza displays. This figure shows, for 6 different patients (**a**–**f**), part of the comparative dashboard allowing for the supervised search of MRD after FlowSOM unsupervised process, in the Kaluza software environment. For each case, the first three plots in the upper row are the respective CD45/SSC biparametric histograms of merged normal bone marrow (NBM), AML patient diagnosis (AML Dg) and follow-up (AML FU). As previously described [15], color codes show the major sub-populations of NBM, all AML Dg cells (powder blue) and all AML FU cells (powder pink). The black color is used to single out the FlowSOM node of interest and thus highlights its backgating on the three CD45/SSC graphs during supervision. The three plots in the middle row display the FREE-FlowSOM MSTs generated as described in the method section, again focused on NBM, AML Dg or AML FU. The same color precedence is used, in particular the black color for the FlowSOM node of interest during supervision. The lower row shows the respective statistical tables corresponding to the characteristics of the black supervised node in NBM, AML Dg and AML FU. Each table displays the normalized MFI ratio of the 9 markers of interest, number of events analyzed, and node events (count, 10^−2^ (%), 10^−3^ or 10^−4^). (**a**) patient #37 is a true positive (TP, i.e., both molecular and flow MRD) case of MRD > 10^−3^; in tube 1, the supervised FlowSOM AML FU node represents a residual minor clone (1.25% in AML FU vs. 4.97% of the cells at diagnosis), however 10 times more abundant than in NBM. (**b**) patient #33 is another TP case of high sensitivity MRD, with tube 2. The FlowSOM AML FU node represents a residual clone <10^−3^ (2x10^−4^), yet predominantly represented at diagnosis (21.23% of the cells). Of note, its CD56+ LAIP, different from normal (DfN), is completely absent in the merged NBM. (**c**) patient #34 is a third TP case, were the supervised AML FU node (tube 2) clearly shows a residual clone with a CD56+ LAIP. The number of residual events detected in this FU is right at the detection limit (17 events for 345 522 total cells). Acquisition of more events would have strengthened this positive MRD, especially since there are virtually no such events in NBM. Moreover, this FU point was clearly positive in tube 1 (not shown). (**d**) patient #26 is a case of false positive (FP) MRD, with undetectable molecular MRD. Yet, the supervised AML FU node (tube 1; 5.7x10^−3^) appears to be the residue of a minor clone present at diagnosis (4.41% of cells), significantly more abundant than in NBM. It could be a population lacking the molecular signature. (**e**) patient #31 is a case of false negative (FN) MRD: despite detectable molecular MRD, AML FU node supervision (tube 1) fails to find a node with similar immunophenotypic characteristics than diagnostic blasts, with a quantity that would be significantly greater than in the NBM. Of note MFC MRD is also negative at this time point with tube 2. (**f**) patient #31 is a case of true negative (TN) MRD where neither molecular nor MFC analyses (tube 2 shown here, but also tube 1) detected residual blasts. Of note, here again, AML Dg blasts displayed a CD56+ LAIP, absent from NBM and undetectable at FU.

For each antibody combination, all listmode (lmd) files were processed using the FlowSOM module (Bioconductor version 3.3.2 with flowsom and flocore packages) integrated to the analysis software Kaluza (Beckman Coulter) as reported [13]. In a first step, compensations were checked and the 12 parameters of each lmd file were normalized. Normalized files of the 19 NBM were then merged and processed to obtain a reference minimal spanning tree (MST) of 100 nodes, called “FROZEN” [13]. The lmd file of merged NBM was then processed together with the normalized diagnosis (AML Dg) and, one by one, each normalized FU (AML FU) lmd file for each patient, in the FlowSOM module. Merge of these three files generated two types of MST. Three (respectively for NBM, Dg and FU) were based on the reference NBM FROZEN MST and assigned Dg and FU populations to the 100 nodes of this NBM MST. A second type of three MST (respectively for NBM, Dg and FU) of 64 nodes was generated, called “FREE”. There, cell populations (nodes) of the normal BM sample were redistributed according to the characteristics of the merged samples of NBM, Dg and FU (Figure 1). The color codes [13] of the reference FROZEN NBM MST were applied automatically to the FREE NBM MST. Both types of Dg and FU MST were respectively colored powder blue and powder pink. These six trees were displayed together. Additionally, according to what has been published previously [15], a selection of nodes of interest (NOI) based on the progenitor area (i.e., “bermudes” as per Arnoulet et al. [27]) was displayed on a different Kaluza sheet. Specific linked gates were defined to track each single node concomitantly in the FREE and NOI displays, with color backgating (in black) on the FROZEN MST and on respective CD45/SSC histograms (Figure 1). As this tracking gate was moved from one node to the other, the respective statistics of mean fluorescence intensity (MFI) for each marker and number of cells in the considered node were displayed for NBM, Dg and FU (Figure 1). This allowed to detect MRD nodes with both the «DfN» or «LAIP» approach.

Based on the criteria retained for NOI definition [15], MRD for any given node was considered positive in a first approach if the percentage of cells in the FU sample was at least twice that of the same node in NBM. As already published also [15], a valid FU node had to represent at least 2% of the blast population. As a consequence, the percentage of cells for this node in NBM had to be less than 1%. Finally, for samples with detectable MRD, the sum of positive nodes for each tube was calculated. The highest value obtained in the more generalist (and congruent with the ELN working group on AML MRD proposal [6]) tube 1 was retained unless MRD was only detected in tube 2.

Four investigators independently examined all FlowSOM processed samples with a specific Kaluza^®^ protocol automatically displaying the graphs and statistics described above, compared their results and reached consensus for all time points. All MFC analyses were performed without knowledge of molecular MRD results.

### 2.4. Statistical Analyses

In order to compare molecular and MFC results, we chose to consider molecular data as gold standard, which allowed, after combination with MFC values, the definition of True Positivity (TP) when both tests were positive, False Positivity (FP) when MFC was positive in the absence of molecular MRD, False Negativity (FN) in the reverse situation of absence of MFC MRD but presence of molecular MRD and True Negativity (TN) when both tests were negative. This was achieved by building a complex matrix analysis (Excel; Microsoft) taking into account MFC NBM and FU levels at all time points compared to the positive or negative status of molecular MRD at the same FU points. The resulting tables were computed with Youden and kappa tests in order to define the best threshold for discriminative sensitivity and specificity of the MCF MRD assessment compared to molecular MRD. This matrix also provided sensitivity, specificity, positive predictive (PPV) and negative predictive (NPV) values.

For correlations between patient outcome and FU data, chi square tests were used (Medcalc Ostend, Belgium).

## 3. Results

### 3.1. MFC MRD Analyses

Data were available for all samples after MFC analysis with both Tube 1 and Tube 2 panels. Therefore, 192 samples and about 1000 potential MRD nodes were examined.

The global strategy adopted to investigate for MFC MRD was, as described above, to set a tracking gate allowing for node-by-node exploration. Nodes of interest were provided by the NOI strategy, completed in some instances by the exploration of a few nearby nodes in the FREE display. Each retained node had to belong to the area of the Dg blast population, confirmed by backgating on the CD45/SSC histogram displayed together with the Dg MST (Figure 1). Cell numbers and percentages of each node in NBM, Dg and FU displays were collected. In no instance did the number of NOI exceed 10 (median and (range) for tubes 1 and 2 respectively 4 (1–9) and 5 (1–10)). *In fine*, the median number of positive nodes were, for tubes 1 and 2, respectively 2 (1–6) and 2 (1–7).

### 3.2. Comparison between MFC and Molecular MRD

Table S2 provides detailed results of MFC and molecular analyses for each FU time-point.

As mentioned in the methods section, the optimal concordance between molecular and MFC MRD was determined using Youden and kappa tests instead of ROC curves. The matrix program was run again after adding the molecular data for each FU time point in terms of positivity or negativity. This demonstrated that the thresholds arbitrarily chosen to establish MCF positivity or negativity could be refined to (i) 1.1% (instead of 1%) for the maximal percentage of NBM cells in a given node and (ii) a FU/NBM ratio of 1.9 instead of 2. This optimized threshold only modified previous results for 3 samples.

**Figure 2:**
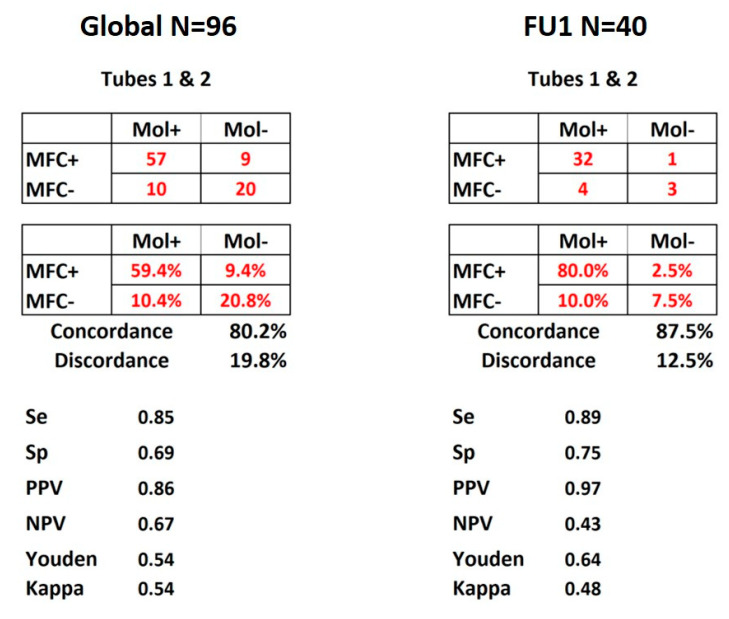
MRD concordance matrices. Display of the results for all 96 FU time points and FU1 of the 40 patients. Both panels show the matrices of numbers and percentages of concordance between molecular and MFC MRD detection according to the combination of tubes 1 and 2. Se: sensitivity, Sp: specificity, PPV: positive predictive value, NPV: negative predictive value. Youden and kappa tests were used and values displayed as well.

As shown in Figure 2, concordance between molecular and FlowSOM MFC detection for the 96 FU time points was 80.2% considering both tubes together. PPV and NPV were respectively of 86% and 67%, with specificity and sensitivity of respectively 85% and 69%. Figure 2 also shows that when the comparison was restricted to the 40 post-induction time-points (FU1, N = 40), the concordance reached 87.5% while PPV and NPV were 97% and 43%, with specificity and sensitivity respectively reaching 89% and 75%.

Figure S1 details the same data separately for tubes 1 and 2 and for the different thresholds based on previous assumptions or after Youden and kappa tests (see above). Figure S2 displays the same comparisons applied respectively to the subgroups of *NPM1*-mutated AML and FAB AML4/5. In spite of the classically reported greater difficulty of identifying MFC MRD in these subsets with monocytic differentiation, similar specificities and sensitivities were obtained as compared to the 96 FU time points or 40 FU1 after unsupervised processing.

### 3.3. Specificities of MFC MRD Analysis

Backgating on CD45/SSC histograms confirmed the similarity between FU and Dg nodes, often disclosing a residual subclone after chemotherapy (Figure 1a,c). Two situations were observed, respectively (i) FREE MST clearly identifying DfN nodes with absolutely no counterpart in NBM (Figure 1b,f), (ii) FU nodes sharing NBM features yet present in a significantly more important size than in NBM according to the thresholds determined (Figure 1a). Of note, the lowest number of FU cells yielding a significant difference with NBM was a cluster of 17 events (Figure 1c).

At variance with what is difficult to identify with classical gating strategies, the FlowSOM unsupervised approach could self-segregate CD56+ and CD56− subclones in the same sample as shown in Figure S3.

**Figure 3:**
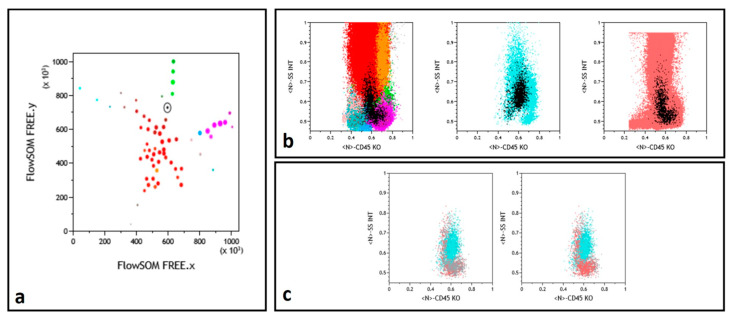
Myelomonocytic regeneration. (**a**) Position of a selected node (round black gate) on the FREE NBM representation, between immature granulocytes (dark red) and monocytes (green). Color codes from ref. [13,15]. (**b**) Backgating (black dots) of this node identifying a regeneration pattern similar in NBM (left) and FU (right) samples and a different population in the middle Dg histogram. (**c**) Selected node CD45/SSC representation with superimposition of the NBM (gray) and FU (powder pink) events while Dg sample (cyan) events are differently backgated.

Finally, in a number of instances, a similar L-shaped pattern was observed for some nodes in the concomitantly tested NBM and FU samples, absent in the concurrent Dg sample (Figure 3). Examination of the immunophenotype of these nodes allowed to conclude that they corresponded to a granulomonocytic maturation pattern, exacerbated in regenerating FU BM, not to be mistaken for MRD.

### 3.4. Correlation between MRD and Clinical Outcome

Examination of the outcome of the 40 patients confirmed CR with negative MFC MRD after induction in only 7 cases vs even less (*n* = 3 cases) with negative molecular MRD (Table 2). These discrepant cases were 3 with persisting *NPM1* mutation and 1 with *RUNX1-RUNX1T1* fusion transcript. Of the 7 patients with negative MFC FU1 at CR completion, 6 are still in CR. One patient (#31) relapsed and died after allogeneic stem cell transplantation (Allo-SCT). For this patient, molecular MRD remained positive until post-AlloSCT. MFC MRD became positive one month before relapse and returned to negativity post-AlloSCT. Conversely, the large majority (N = 29) of CR patients retained both positive post-induction MFC and molecular MRD. Induction failure (more than 5% of blasts in BM at the end of aplasia), noted for 4 patients, correlated with positive MFC MRD for all and with positive molecular MRD for 3. The patient with negative molecular MRD (#9) likely had lost the molecular target (*NPM1* mutation) and this discrepancy constitutes a questionable FP MFC MRD.

All in all, with 17.5% of CR patients showing negative MRD, MFC could be more sensitive to predict early chemosensitivity than molecular MRD detection (7.5%) at FU1. Indeed, by testing transcripts, molecular methods may identify fewer cells with large amounts of RNA. Only follow-up of the patients and larger series will ultimately allow to conclude about the best predictor.

## 4. Discussion

This study demonstrates that an unsupervised analysis of MFC data allows to explore MRD in AML patients with a high confidence in the populations observed. The setting of this work was challenging, since it was decided to include only patients with a possible concomitant molecular FU, known to represent only a proportion of AML patients. Moreover, *NPM1* mutations are notably associated with myelomonocytic differentiation [28]. This is a real challenge as there is a continuum between monocytic blasts and normal monocytes at diagnosis and normal reconstitution at FU is difficult to dissociate from remaining tumor cells. No specific strategy has been published in the literature regarding these specific populations [29]. Here, the position of MRD nodes was clearly related to monocytic differentiation but the concomitant comparison with NBM greatly facilitated their discrimination. This is consistent with the few examples in the literature of separating blasts from hematopoiesis with non-supervised strategies in mass cytometry [14,30]. The power of the combination of FlowSOM and Kaluza^®^ allows to apply nodes assignment to a mixed population of NBM, Dg and FU lmds which are thus treated in exactly the same way, then to concomitantly display the result of the three MST side by side. This is clearly completely different from the reference to a few (usually not shown) NBM samples reported in many MRD studies. One of the first examples of this indirect comparison was provided by the notion of “empty boxes” [31,32]. In this strategy, MFC analysis is set-up so as to draw gates in the analysis of NBM (with full and empty boxes) and, in a second step, apply the same template to Dg or FU samples. This has also been a strategy retained for example by Kern et al. [33]. Of note, this imposes to use highly harmonized set-ups as well as exactly the same panels of antibodies for the NBM and patient’s samples. This has been demonstrated to be feasible, especially with the very stable current instruments and validated strategies [24,34]. However, by definition, LAIP cannot be compared to NBM in such separated analyses in the majority of cases.

Another advantage of the FlowSOM strategy is of course that the software identifies populations that would have been difficult to select through sequential gating yet can be perfectly characterized during the supervised part of the analysis. To our great satisfaction, FlowSOM allowed to straightforwardly demonstrate concepts taken into account as important in the ELN proposal [6]. As shown in the examples provided, some nodes validated the DfN concept, through blast populations completely absent in the concomitantly analyzed NBM. LAIPs were also self-selected by the algorithm, with the amazing evidence of, in a single Dg sample, subclones carrying or not the CD56 LAIP. This self-selection is liable to take into account antigenic modulations that may escape the classical LAIP approach. Finally, the recurrent question of regenerating BM, questioned when considering comparison of blasts with NBM, became self-illustrated in the evidence of matching hematopoietic differentiation patterns in an FU node and its NBM counterpart. This specific pattern, but also the systematic backgating of the nodes examined on a CD45/SSC histogram, reinforce the importance of this representation [35,36].

FlowSOM concomitant analysis of NBM, Dg and FU further allowed to approach the critical issue of significant threshold. As established in the definition of the FlowSOM scripts [15], we chose to consider nodes displaying at least twice the percentage of cells as the corresponding NBM node. This was confirmed here by the statistical analysis comparing MFC and molecular final data, which yielded a 1.9 ratio between FU and NBM as the most pertinent.

In our series, the lowest number of FU cells complying with this criterion was 17 cells. This confirms the global opinion of considering clusters of 10–20 cells for limit of detection (LOD) [6]. Of course, this is limited by the number of acquired cells. As we used retrospective data, but also because cell number limitations are inherent to the samples, some results would certainly have been consolidated by the acquisition of more events. Nonetheless, the robustness of this 15–20 events (or perhaps even 10 in case of truly DfN) validates that the current accepted 10^−3^ threshold [6] can be improved down to 10^−5^ upon acquisition of a sufficient number of cells for negative samples.

Another limitation of our study is that we chose molecular MRD as gold standard, raising the question of the significance of FP data, i.e., positive MFC MRD and negative molecular MRD. Nevertheless, the concordance obtained in the different conditions tested (all time points, FU1 only, NPM1 only, AML4/5 only) appears quite satisfactory. These types of comparison have been reported in a few cases in the literature with classical MFC analyses. Kern et al. [33], in a cohort of 144 patients with 372 FU samples, reported a concordance of 61% with equivalent proportions of FP and FN samples. In 2012, Rossi et al. [37], for 23 patients at FU1, obtained a specificity of 53.8% and a sensitivity of 80% when comparing molecular and MFC MRD assessments. More recently, the HOVON/SAKK AML 42A study [9], in a series of 340 patients, compared a tailored NGS panel and MFC performed according to Terwijn et al. [38]. This study displayed 69.1% of concordance and respectively 64 FP and 41 FN cases. Finally, in the GIMEMA AML1310 trial [39], a concordance of 43% was reported between molecular and MFC MRD assessment for 60 FU1 samples. However, only patients with double positive MRD displayed a significantly lower OS and PFS. Of interest, our evaluation in a phenotypically difficult series of AML yielded much better results, probably comforting the expected superiority of unsupervised definition of cell subsets. Applied to AML patients, even in the absence of a molecular marker, the unsupervised strategy reported here would allow for MFC MRD assessment in all cases. This has to be confirmed in an independent validation study.

## 5. Conclusions

Overall, this study demonstrates the power of the combined unsupervised FlowSOM and Kaluza^®^ software, to evaluate AML MRD. In the challenging set-up of this study, that only considered patients with molecular MRD targets, MFC MRD assessment stood out as a useful monitoring tool. It is thus likely that FlowSOM-assisted MFC MRD assessment, using unsupervised treatment of .fcs files, will be applicable to all AML patients and provide clinicians with useful hints for treatment options.

## Figures and Tables

**Table 1:** Patient characteristics.

Characteristic	Value or Numbers
Age in years	
Median	54
Range	21–70 (+1 7yo child)
Gender	
Male	24
Female	16
Type of AML (WHO)	
AML with recurrent cytogenetic abnormalities	10
AML with BCR-ABL	2
AML with myelodysplasia-related changes	6
AML with mutated NPM1 (without maturation)	6
AML with mutated NPM1 (with maturation)	3
AML with mutated NPM1 (myelomonocytic)	8
AML with mutated NPM1 (monoblastic/monocytic)	5
Molecular markers used for mMRD	
*NPM1 A*	24
*NPM1 B*	3
*NPM1 D*	2
*RUNX1-RUNX1T1*	6
*CBFB-MYH11*	4
*BCR-ABL*	1
Follow-up points	
FU1 (post induction)	40
Total FU points 1, 2, 3, 4, 5, 6	12, 13, 6, 6, 2, 1

Italics: gene names.

**Table 2:** Clinical correlations at FU1.

MRD Status	Induction Failure N (%)	Complete Remission N (%)
MFC MRD		
Negative	0 (0)	7 (17.5)
Positive	4 (10)	29 (72.5)
Molecular MRD		
Negative	1 (2.5)	3 (7.5)
Positive	3 (7.5)	33 (82.5)

## Data Availability

The data presented in this study are available on request from the corresponding author. The data are not publicly available due to patient information in .fcs files.

## Supplementary Materials

The following are available online at https://www.mdpi.com/2072-6694/13/4/629/s1, Figure S1: Display of the results for all 96 time points and FU1 of the 40 patients with different threshold parameters. Figure S2: Display of the results for two different subgroups of patients. Top panel: Patients with NPM1 mutation only according to all time points (N = 60) or FU1 only (N = 29). Figure S3: Unsupervised delineation of two adjacent but distinct nodes identifying subclones with different LAIP at diagnosis, respectively CD56-positive (top) and CD56-negative (bottom). Table S1: Complement on patient characteristics. Table S2: Detailed point by point patient status and results of MRD Assessment.

## References

[1] Döhner H., Estey E., Grimwade D., Amadori S., Appelbaum F.R., Büchner T., Dombret H., Ebert B.L., Fenaux P., Larson R.A. (2017). Diagnosis and management of AML in adults: 2017 ELN recommendations from an international expert panel. Blood.

[2] Burnett A.K., Goldstone A., Hills R.K., Milligan D.W., Prentice A., Yin J., Wheatley K., Hunter A., Russell N. (2013). Curability of patients with acute myeloid leukemia who did not undergo transplantation in first remission. J. Clin. Oncol..

[3] Komanduri K.V., Levine R.L. (2016). Diagnosis and therapy of acute myeloid leukemia in the era of molecular risk stratification. Annu. Rev. Med..

[4] Cheson B.D., Bennett J.M., Kopecky K.J., Büchner T., Wilman C.L., Estey E.H., Schiffer C.A., Döhner H., Tallman M.S., Lister T.A. (2003). Revised recommendations of the International Working Group for Diagnosis, Standardization of Response Criteria, Treatment Outcomes, and Reporting Standards for Therapeutic Trials in Acute Myeloid Leukemia. J. Clin. Oncol..

[5] Voso M.T., Ottone T., Lavorgna S., Venditti A., Maurillo L., Lo Coco F., Buccisano F. (2019). MRD in AML: The role of new techniques. Front. Oncol..

[6] Schuurhuis G.J., Heuser M., Freeman S., Béné M.C., Buccisano F., Cloos J., Grimwade D., Haferlach T., Hills R.K., Hourigan C.S. (2018). Minimal/measurable residual disease in AML: A consensus document from the European LeukemiaNet MRD Working Party. Blood.

[7] Grimwade D., Freeman S.D. (2014). Defining minimal residual disease in acute myeloid leukemia: Which platforms are ready for “prime time”?. Blood.

[8] Papaemmanuil E., Gerstung M., Bullinger L., Gaidzik V.I., Paschka P., Roberts N., Potter N.E., Heuser M., Thol F., Bolli N. (2016). Genomic Classification and Prognosis in Acute Myeloid Leukemia. N. Engl. J. Med..

[9] Jongen-Lavrencic M., Grob T., Hanekamp D., Kevlaars F.G., al Hinai A., Zeilemaker A., Erpelinck-Verschueren C.A.J., Gradowska P.L., Meijer R., Cloos J. (2018). Molecular minimal residual disease in acute myeloid leukemia. N. Engl. J. Med..

[10] Zeijlemaker W., Gratama J.W., Schuurhuis G.J. (2014). Tumor heterogeneity makes AML a “moving target” for detection of residual disease. Cytom. B Clin. Cytom..

[11] Weber L.M., Robinson M.D. (2016). Comparison of clustering methods for high-dimensional single-cell flow and mass cytometry data. Cytom. Part A.

[12] Van Gassen S., Callebaut B., Van Helden M.J., Lambrecht B.N., Demeester P., Dhaene T., Saeys Y. (2015). FlowSOM: Using self-organizing maps for visualization and interpretation of cytometry data. Cytom. Part A.

[13] Lacombe F., Dupont B., Lechevalier N., Vial J.P., Béné M.C. (2019). Innovation in flow cytometry analysis: A new paradigm delineating normal or diseased bone marrow subsets through machine learning. HemaSphere.

[14] Amir E.D., Davis K.L., Tadmor M.D., Simonds E.F., Levine J.H., Bendall S.C., Shenfeld D.K., Krishnaswamy S., Nolan G.P., Pe’er D. (2013). viSNE enables visualization of high dimensional single-cell data and reveals phenotypic heterogeneity of leukemia. Nat. Biotechnol..

[15] Lacombe F., Lechevalier N., Vial J.P., Béné M.C. (2019). An R-derived FlowSOM process to analyze unsupervised clustering of normal and malignant human bone marrow classical flow cytometry data. Cytom. Part A.

[16] Spitzer M.H., Nolan G.P. (2016). Mass cytometry: Single cells, many features. Cell.

[17] Van der Maaten L., Hinton G. (2008). Visualizing Data using t-SNE. J. Mach. Learn. Res..

[18] Levine J.H., Simonds E.F., Bendall S.C., Davis K.L., Amir E.D., Tadmor M.D., Litvin O., Fienberg H.G., Jager A., Zunder E.R. (2015). Data-driven phenotypic dissection of AML reveals progenitor-like cells that correlate with prognosis. Cell.

[19] Mair F., Hartmann F.J., Mrdjen D., Tosevski V., Krieg C., Becher B. (2016). The end of gating? An introduction to automated analysis of high dimensional cytometry data. Eur. J. Immunol..

[20] Swerdlow S., Campo E., Harris N., Jaffz E.S., Pileri S.A., Stein H., Thiele J. (2017). WHO Classification of Tumours of Haematopoietic and Lymphoid Tissues.

[21] Bertoli S., Bories P., Béné M.C., Daliphard S., Lioure B., Pigneux A., Vey N., Delaunay J., Leymarie V., Luquet I. (2014). Prognostic impact of day 15 blast clearance in risk-adapted remission induction chemotherapy for younger patients with acute myeloid leukemia: Long-term results of the multicenter prospective LAM-2001 trial by the GOELAMS study group. Haematologica.

[22] Béné M.C., Nebe T., Bettelheim P., Buldini B., Bumbea H., Kern W., Lacombe F., Lemez P., Marinov I., Matutes E. (2011). Immunophenotyping of acute leukemia and lymphoproliferative disorders: A consensus proposal of the European LeukemiaNet Work Package 10. Leukemia.

[23] Collective Publication (2018). Panel proposal for the immunophenotypic diagnosis of hematological malignancies. A collaborative consensus from the groupe d’Etude Immunologique des Leucémies (GEIL). Cytom. B Clin. Cytom..

[24] Lacombe F., Bernal E., Bloxham D., Couzens S., Della Porta M., Johansson U., Kern W., Macey M., Matthes T., Morilla R. (2016). Harmonemia: A universal strategy for flow cytometry immunophenotyping—A European LeukemiaNet WP10 study. Leukemia.

[25] Krönke J., Schlenk R.F., Jensen K.-O., Tschürtz F., Corbacioglu A., Gaudzik V.I., Paschka P., Onken S., Eiwen K., Habdank M. (2011). Monitoring of minimal residual disease in NPM1-mutated acute myeloid leukemia: A study from the German-Austrian acute myeloid leukemia study group. J. Clin. Oncol..

[26] Gabert J., Beillard E., van der Velden V.H.J., Bi W., Grimwade D., Pallisgaard N., Barbany G., Cazzaniga G., Cayuela J.M., Cavé H. (2003). Standardization and quality control studies of “real-time” quantitative reverse transcriptase polymerase chain reaction of fusion gene transcripts for residual disease detection in leukemia—A Europe Against Cancer program. Leukemia.

[27] Arnoulet C., Béné M.C., Durrieu F., Feuillard J., Fossat C., Husson B., Jouault H., Maynadié M., Lacombe F. (2010). Four- and five-color flow cytometry analysis of leukocyte differentiation pathways in normal bone marrow: A reference document based on a systematic approach by the GTLLF and GEIL. Cytom. B Clin. Cytom..

[28] Bain B.J., Béné M.C. (2019). Morphological and immunophenotypic clues to the WHO categories of acute myeloid leukaemia. Acta Haematol..

[29] Al-Mawali A., Gillis D., Lewis I. (2009). The role of multiparameter flow cytometry for detection of minimal residual disease in acute myeloid leukemia. Am. J. Clin. Pathol..

[30] Diggins K.E., Ferrell P.B., Irish J.M. (2015). Methods for discovery and characterization of cell subsets in high dimensional mass cytometry data. Methods.

[31] Campana D., Coustan-Smith E. (1999). Detection of minimal residual disease in acute leukemia by flow cytometry. Cytometry.

[32] Lucio P., Gaipa G., van Lochem E.G., van Wering E.R., Porwit-MacDonald A., Faria T., Bjorklund E., Biondi A., van den Beemd M.W.M., Baars E. (2001). BIOMED-I concerted action report: Flow cytometric immunophenotyping of precursor B-ALL with standardized triple-stainings. BIOMED-1 Concerted Action Investigation of Minimal Residual Disease in Acute Leukemia: International Standardization and Clinical Evaluation. Leukemia.

[33] Kern W., Schoch C., Haferlach T., Schnittger S. (2005). Monitoring of minimal residual disease in acute myeloid leukemia. Crit. Rev. Oncol. Hematol..

[34] Kalina T., Flores-Montero J., van der Velden V.H.J., Martin-Ayuso M., Böttcher S., Ritgen M., De Almeida J.G., Lhermitte L., Asnafi V., Mendonca A. (2012). EuroFlow standardization of flow cytometer instrument settings and immunophenotyping protocols. Leukemia.

[35] Lacombe F., Durrieu F., Briais A., Dumain P., Belloc F., Bascans E., Reiffers J., Boisseau M.R., Bernard P. (1997). Flow cytometry CD45 gating for immunophenotyping of acute myeloid leukemia. Leukemia.

[36] Kern W., Haferlach C., Haferlach T., Schnittger S. (2008). Monitoring of minimal residual disease in acute myeloid leukemia. Cancer.

[37] Rossi G., Carella A.M., Minervini M.M., Savino L., Fontana A., Pellegrini F., Greco M.M., Merla E., Quarta G., Loseto G. (2013). Minimal residual disease after allogeneic stem cell transplant: A comparison among multiparametric flow cytometry, Wilms tumor 1 expression and chimerism status (Complete chimerism versus Low Level Mixed Chimerism) in acute leukemia. Leuk. Lymphoma.

[38] Terwijn M., van Putten W.L.J., Kelder A., van der Velden V., Brooimans R.A., Pabst T., Maertens J., Boecx N., de Greef G.E., Valk P.J.M. (2013). High prognostic impact of flow cytometric minimal residual disease detection in acute myeloid leukemia: Data from the HOVON/SAKK AML 42A study. J. Clin. Oncol..

[39] Venditti A., Piciocchi A., Candoni A., Melillo L., Calafiore V., Cairoli R., De Fabritiis P., Storti G., Salutari P., Lanza F. (2019). GIMEMA AML1310 trial of risk-adapted, MRD-directed therapy for young adults with newly diagnosed acute myeloid leukemia. Blood.

