# Supplementary material for: Unsupervised Flow Cytometry Analysis Allows for an Accurate Identification of Minimal Residual Disease Assessment in Acute Myeloid Leukemia
Source: Cancers (Basel). 2021 Feb 5;13(4):629. doi: 10.3390/cancers13040629 (PMC7914957; doi:10.3390/cancers13040629)
Supplement: Supplementary file 1 [file cancers-13-00629-s001.pdf]

cancers

Supplemental Material

Unsupervised Flow Cytometry Analysis Allows for an Accurate Identification of Minimal Residual Disease Assessment in Acute Myeloid Leukemia

Jean Philippe Vial, Nicolas Lechevalier, Francis Lacombe, Pierre-Yves Dumas, Audrey Bidet, Thibaut Leguay, François Vergez, Arnaud Pigneux and Marie C Béné

Table S1. Complement on patient characteristics

Patient (#)	WHO classification	Molecular markers used for MRD	FLT3 status	Karyotype	Number of FU
1	AML with mutated NPM1 (myelomonocytic)	*NPM1 A*	ITD	normal	2
2	AML with mutated NPM1 (monoblastic/monocytic)	*NPM1 A*	WT	normal	4
3	AML with mutated NPM1 (myelomonocytic)	*NPM1 A*	WT	normal	3
4	AML with BCR-ABL	*NPM1 A*	WT	t(9;22)	1
5	AML with mutated NPM1 (myelomonocytic)	*NPM1 A*	WT	normal	2
6	AML with mutated NPM1 (myelomonocytic)	*NPM1 A*	WT	normal	1
7	AML with mutated NPM1 (with maturation)	*NPM1 A*	WT	normal	2
8	AML with mutated NPM1 (myelomonocytic)	*NPM1 A*	ITD	normal	2
9	AML with mutated NPM1 (monoblastic/monocytic)	*NPM1 A*	WT	complex	2
10	AML with mutated NPM1 (myelomonocytic)	*NPM1 A*	ITD	normal	1
11	AML with mutated NPM1 (without maturation)	*NPM1 A*	ITD	normal	1
12	AML with mutated NPM1 (without maturation)	*NPM1 A*	ITD	normal	1
13	AML with mutated NPM1 (monoblastic/monocytic)	*NPM1 A*	WT	normal	1
14	AML with mutated NPM1 (myelomonocytic)	*NPM1 A*	WT	normal	1
15	AML with mutated NPM1 (without maturation)	*NPM1 A*	ITD	normal	3
16	AML with mutated NPM1 (myelomonocytic)	*NPM1 A*	ITD	normal	2
17	AML with mutated NPM1 (with maturation)	*NPM1 A*	WT	normal	2
18	AML with mutated NPM1 (monoblastic/monocytic)	*NPM1 A*	TKD	normal	4
19	AML with mutated NPM1 (with maturation)	*NPM1 A*	ITD	trisomy 8	4
20	AML with mutated NPM1 (without maturation)	*NPM1 A*	ITD	normal	2
21	AML with mutated NPM1 (without maturation)	*NPM1 A*	WT	normal	4
22	AML with mutated NPM1 (without maturation)	*NPM1 A*	WT	normal	4
23	AML with mutated NPM1 (without maturation)	*NPM1 A*	ITD	normal	3
24	AML with mutated NPM1 (myelomonocytic)	*NPM1 A*	ITD	normal	1
25	AML with mutated NPM1 (monoblastic/monocytic)	*NPM1 B*	WT	normal	2
26	AML with mutated NPM1 (monoblastic/monocytic)	*NPM1 B*	ITD	normal	2
27	AML with mutated NPM1 (monoblastic/monocytic)	*NPM1 B*	TKD	normal	1
28	AML with mutated NPM1 (monoblastic/monocytic)	*NPM1 D*	ITD	normal	1
29	AML with mutated NPM1 (myelomonocytic)	*NPM1 D*	WT	normal	1
30	AML with recurrent cytogenetic abnormalities	*RUNX1-RUNXT1*	NA	t(8;21)	2
31	AML with recurrent cytogenetic abnormalities	*RUNX1-RUNXT1*	NA	t(8;21)	5
32	AML with recurrent cytogenetic abnormalities	*RUNX1-RUNXT1*	NA	t(8;21)	1
33	AML with recurrent cytogenetic abnormalities	*RUNX1-RUNXT1*	NA	t(8;21)	3

34	AML with recurrent cytogenetic abnormalities	*RUNX1-RUNXT1*	NA	t(8;21)	5
35	AML with recurrent cytogenetic abnormalities	*RUNX1-RUNXT1*	NA	t(8;21)	4
36	AML with recurrent cytogenetic abnormalities	*CBFB-MYH11*	NA	inv(16)	6
37	AML with recurrent cytogenetic abnormalities	*CBFB-MYH11*	NA	inv(16)	2
38	AML with recurrent cytogenetic abnormalities	*CBFB-MYH11*	NA	inv(16)	3
39	AML with recurrent cytogenetic abnormalities	*CBFB-MYH11*	NA	inv(16)	3
40	AML with BCR-ABL	*BCR-ABL*	NA	t(9;22)	2
					96

Table S2. Detailed point by point patient status and results of MRD Assessment.
FU: Follow-up; d: days after diagnosis; FS-SORN: FlowSOM sum of retained nodes; CR: complete remission.

#	FU (d)	Disease status	Gene	Gene/ABL (%)	Molecular MRD	FS-SORN (%)	MFC-MRD
1	diag	AML4	*NPM1 A*	892		63.6	
	39	CR		0.0001	-	0	-
	530	CR		0.0001	-	0	-
2	diag	AML5	*NPM1 A*	1120		56.2	
	74	CR		0.0072	+	1.00	+
	162	CR		0.0001	-	0	-
	428	relapse		1897	+	2.14	+
	470	CR		0.0010	-	0	-
3	diag	AML4	*NPM1 A*	1309		52.68	
	38	CR		0.7174	+	1.55	+
	407	relapse		444	+	6.85	+
	837	CR		0.006	+	5.00	+
4	diag	AML2	*NPM1 A*	710		40.16	
	28	CR		0.1029	+	0.23	+
5	diag	AML MRC	*NPM1 A*	6000		58.59	
	53	CR		0.0142	+	2.7	+
	217	CR		0.0051	+	0.23	+
6	diag	AML4	*NPM1 A*	1317		73.37	
	39	CR		0.1233	+	7.37	+
7	diag	AML2	*NPM1 A*	1337		45.44	
	38	CR		8.4	+	0.48	+
	148	CR		7.57	+	2.36	+
8	diag	AML4	*NPM1 A*	1623		65.9	
	36	refractory		555	+	8.50	+
	198	relapse		3337	+	32.53	+
9	diag	AML5	*NPM1 A*	1374		40.19	
	38	refractory		0.0001	-	3.87	+
	48	refractory		0.0001	-	2.33	+
10	diag	AML4	*NPM1 A*	3910		50.45	
	44	refractory		40	+	0.32	+
11	diag	AML1	*NPM1 A*	3647		63.05	
	47	CR		0.4539	+	0.13	+
12	diag	AML5	*NPM1 A*	2457		81.71	
	38	CR		0.8026	+	1.47	+
13	diag	AML5	*NPM1 A*	1770		31.47	
	72	CR		0.0289	+	1.60	+
14	diag	AML5	*NPM1 A*	2533		41.06	
	49	CR		0.0001	-	0	-
15	diag	AML1	*NPM1 A*	2718		74.69	
	35	CR		44	+	0	-
	68	CR		0.001	-	0	-
	209	CR		0.001	-	0	-
16	diag	AML4	*NPM1 A*	2253		48.33	
	70	CR		0.0317	+	0	-

	149	CR		0.0251	+	0	-
17	diag	AML2	*NPM1 A*	335		53.01	
	52	CR		0.001	-	0	-
	91	CR		0.001	-	0.77	+
18	diag	AML5	*NPM1 A*	846		81.13	
	39	CR		0.0685	+	1.85	+
	74	CR		0.040	+	4.76	+
	111	CR		0.001	-	9.71	+
	256	CR		0.001	-	5.30	+
19	diag	AML2	*NPM1 A*	1700		65.72	
	43	CR		0.770	+	1.20	+
	77	CR		0.000	-	0	-
	112	CR		0.000	-	2.16	+
	146	CR		0.000	-	0	-
20	diag	AML5	*NPM1 A*	970		81.14	
	36	CR		0.5742	+	0.09	+
	76	CR		0.001	-	0	-
21	diag	AML1	*NPM1 A*	6220		45.83	
	39	CR		0.088	+	1.43	+
	77	CR		0.001	-	0	-
	112	CR		0.001	-	0	-
	145	CR		0.001	-	0	-
22	diag	AML1	*NPM1 A*	690		69.56	
	36	CR		2.5	+	1.86	+
	71	CR		0.04	+	3.29	+
	106	CR		0.000	-	3.53	+
	140	CR		0.02	+	1.11	+
23	diag	AML1	*NPM1 A*	2176		76.27	
	61	CR		0.038	+	0.96	+
	95	CR		0.001	-	0	-
	138	CR		0.001	-	2.06	+
24	diag	AML MRC	*NPM1 A*	2665		50.37	
	49	CR		0.0061	+	0.23	+
25	diag	AML5	*NPM1 B*	776		54.7	
	40	CR		0.0117	+	1.52	+
	503	CR		0.0001	-	0	-
26	diag	AML5	*NPM1 B*	3156		78.44	
	35	CR		0.9153	+	4.76	+
	242	CR		0.0001	-	3.01	+
27	diag	AML5	*NPM1 B*	2356		41.44	
	35	CR		0.238	+	1.65	+
28	diag	AML5	*NPM1 D*	2015		78.21	
	39	CR		0.1841	+	0	-
29	diag	AML4	*NPM1 D*	1705		20.18	
	35	CR		3.832	+	3.53	+
30	diag	AML2	*RUNX1-RUNX1T1*	209		30.26	
	39	CR		2.938	+	2.07	+
	190	relapse		488	+	9.17	+

31	diag	AML2	*RUNX1-RUNX1T1*	329		60.54	
	44	CR		2.525	+	0	-
	73	CR		0.1989	+	0	-
	167	CR		36	+	0.17	+
	199	relapse		147	+	20.62	+
	341	CR		0.0001	-	0	-
32	diag	AML2	*RUNX1-RUNX1T1*	348		43.62	
	39	CR		5.567	+	0.63	+
33	diag	AML2	*RUNX1-RUNX1T1*	1262		82.9	
	37	CR		29	+	0.82	+
	104	CR		1.861	+	0.96	+
	125	CR		1.412	+	1.34	+
34	diag	AML2	*RUNX1-RUNX1T1*	1372		58.89	
	38	CR		2.952	+	2.08	+
	75	CR		0.725	+	1.92	+
	115	CR		0.073	+	3.29	+
	144	CR		0.115	+	0	-
	322	CR		9.6	+	0.65	+
35	diag	AML2	*RUNX1-RUNX1T1*	584		31.13	
	33	CR		0.96	+	1.98	+
	68	CR		0.145	+	1.26	+
	103	CR		0.0135	+	0.23	+
	139	CR		0.0001	-	0	-
36	diag	AML4	*CBFB-MYH11*	243		55.93	
	42	CR		0.0137	+	3.60	+
	78	CR		0.022	+	0	-
	463	CR		0.061	+	0	-
	492	relapse		0.012	+	0.38	+
	533	CR		0.012	+	0.67	+
	654	relapse		0.0001	-	0	-
37	diag	AML4	*CBFB-MYH11*	107		62.94	
	35	CR		0.204	+	1.43	+
	97	CR		0.21	+	2.11	+
38	diag	AML4	*CBFB-MYH11*	ND		43.40	
	39	CR		0.008	+	1.93	+
	181	CR		0.01	+	0	-
	277	CR		0.0001	-	0	-
39	diag	AML4	*CBFB-MYH11*	84		20.60	
	40	CR		0.11	+	2.03	+
	74	CR		0.055	+	1.16	+
	111	CR		0.017	+	0.89	+
40	diag	A-CML	*BCR-ABL*	127		22.41	
	63	refractory		84	+	6.51	+
	91	CR		82	+	3.22	+

Settings:
NBM max cell 1%
Ratio FU/NBM ≥2

Global

	Tube 1 Mol+	Tube 1 Mol-	Tube 2 Mol+	Tube 2 Mol-	Tubes 1 & 2 Mol+	Tubes 1 & 2 Mol-
MFC+	38	7	46	7	54	9
MFC-	29	22	21	22	13	20
MFC+	39.6%	7.3%	47.9%	7.3%	56.3%	9.4%
MFC-	30.2%	22.9%	21.9%	22.9%	13.5%	20.8%

	Tube 1	Tube 2	Tubes 1 & 2
Concordance	62.5%	70.8%	77.1%
Discordance	37.5%	29.2%	22.9%
Se	0.57	0.69	0.81
Sp	0.76	0.76	0.69
PPV	0.84	0.87	0.86
NPV	0.43	0.51	0.61
Youden	0.33	0.45	0.50
Kappa	0.27	0.39	0.48

FU1

	Tube 1 Mol+	Tube 1 Mol-	Tube 2 Mol+	Tube 2 Mol-	Tubes 1 & 2 Mol+	Tubes 1 & 2 Mol-
MFC+	23	1	24	1	30	1
MFC-	13	3	12	3	6	3
MFC+	57.5%	2.5%	60.0%	2.5%	75.0%	2.5%
MFC-	32.5%	7.5%	30.0%	7.5%	15.0%	7.5%

	Tube 1	Tube 2	Tubes 1 & 2
Concordance	65.0%	67.5%	82.5%
Discordance	35.0%	32.5%	17.5%
Se	0.64	0.67	0.83
Sp	0.75	0.75	0.75
PPV	0.96	0.96	0.97
NPV	0.19	0.20	0.33
Youden	0.39	0.42	0.58
Kappa	0.17	0.19	0.38

Settings:
NBM max cell 1.1%
Ratio FU/NBM ≥1.9

Global

	Tube 1 Mol+	Tube 1 Mol-	Tube 2 Mol+	Tube 2 Mol-	Tubes 1 & 2 Mol+	Tubes 1 & 2 Mol-
MFC+	40	8	48	7	57	9
MFC-	27	21	19	22	10	20
MFC+	41.7%	8.3%	50.0%	7.3%	59.4%	9.4%
MFC-	28.1%	21.9%	19.8%	22.9%	10.4%	20.8%

	Tube 1	Tube 2	Tubes 1 & 2
Concordance	63.5%	72.9%	80.2%
Discordance	36.5%	27.1%	19.8%
Se	0.60	0.72	0.85
Sp	0.72	0.76	0.69
PPV	0.83	0.87	0.86
NPV	0.44	0.54	0.67
Youden	0.32	0.48	0.54
Kappa	0.27	0.43	0.54

FU1

	Tube 1 Mol+	Tube 1 Mol-	Tube 2 Mol+	Tube 2 Mol-	Tubes 1 & 2 Mol+	Tubes 1 & 2 Mol-
MFC+	25	1	25	1	32	1
MFC-	11	3	11	3	4	3
MFC+	62.5%	2.5%	62.5%	2.5%	80.0%	2.5%
MFC-	27.5%	7.5%	27.5%	7.5%	10.0%	7.5%

	Tube 1	Tube 2	Tubes 1 & 2
Concordance	70.0%	70.0%	87.5%
Discordance	30.0%	30.0%	12.5%
Se	0.69	0.69	0.89
Sp	0.75	0.75	0.75
PPV	0.96	0.96	0.97
NPV	0.21	0.21	0.43
Youden	0.44	0.44	0.64
Kappa	0.21	0.21	0.48

Figure S1: Display of the results for all 96 time points and FU1 of the 40 patients with different threshold parameters. Top panel: settings derived from published data (15). Bottom panel: optimized setting according to Youden and kappa tests (as in Figure 2). Matrices of numbers and percentages of concordance between molecular and MFC MRD detection are shown for tube 1, tube 2 and the combination of both tubes. Se: sensitivity, Sp: specificity, PPV: positive predictive value, NPV: negative predictive value. Youden and kappa tests were used and values displayed as well.

Global NPM

Tube 1

	Mol+	Mol-
MFC+	23	8
MFC-	12	17

	Mol+	Mol-
MFC+	38.3%	13.3%
MFC-	20.0%	28.3%

Concordance 66.7%
Discordance 33.3%

Se	0.66
Sp	0.68
PPV	0.74
NPV	0.59
Youden	0.34
Kappa	0.33

Tube 2

	Mol+	Mol-
MFC+	25	7
MFC-	10	18

	Mol+	Mol-
MFC+	41.7%	11.7%
MFC-	16.7%	30.0%

Concordance 71.7%
Discordance 28.3%

Se	0.71
Sp	0.72
PPV	0.78
NPV	0.64
Youden	0.43
Kappa	0.43

Tubes 1 & 2

	Mol+	Mol-
MFC+	31	9
MFC-	4	16

	Mol+	Mol-
MFC+	51.7%	15.0%
MFC-	6.7%	26.7%

Concordance 78.3%
Discordance 21.7%

Se	0.89
Sp	0.64
PPV	0.78
NPV	0.80
Youden	0.53
Kappa	0.54

FU1 NPM

Tube 1

	Mol+	Mol-
MFC+	17	1
MFC-	8	3

	Mol+	Mol-
MFC+	58.6%	3.4%
MFC-	27.6%	10.3%

Concordance 69.0%
Discordance 31.0%

Se	0.68
Sp	0.75
PPV	0.94
NPV	0.27
Youden	0.43
Kappa	0.25

Tube 2

	Mol+	Mol-
MFC+	16	1
MFC-	9	3

	Mol+	Mol-
MFC+	55.2%	3.4%
MFC-	31.0%	10.3%

Concordance 65.5%
Discordance 34.5%

Se	0.64
Sp	0.75
PPV	0.94
NPV	0.25
Youden	0.39
Kappa	0.21

Tubes 1 & 2

	Mol+	Mol-
MFC+	22	1
MFC-	3	3

	Mol+	Mol-
MFC+	75.9%	3.4%
MFC-	10.3%	10.3%

Concordance 86.2%
Discordance 13.8%

Se	0.88
Sp	0.75
PPV	0.96
NPV	0.50
Youden	0.63
Kappa	0.52

Global AML 4-5

Tube 1

	Mol+	Mol-
MFC+	18	5
MFC-	15	9

	Mol+	Mol-
MFC+	38.3%	10.6%
MFC-	31.9%	19.1%

Concordance 57.4%
Discordance 42.6%

Se	0.55
Sp	0.64
PPV	0.78
NPV	0.38
Youden	0.19
Kappa	0.16

Tube 2

	Mol+	Mol-
MFC+	25	3
MFC-	8	11

	Mol+	Mol-
MFC+	53.2%	6.4%
MFC-	17.0%	23.4%

Concordance 76.6%
Discordance 23.4%

Se	0.76
Sp	0.79
PPV	0.89
NPV	0.58
Youden	0.54
Kappa	0.49

Tubes 1 & 2

	Mol+	Mol-
MFC+	27	5
MFC-	6	9

	Mol+	Mol-
MFC+	57.4%	10.6%
MFC-	12.8%	19.1%

Concordance 76.6%
Discordance 23.4%

Se	0.82
Sp	0.64
PPV	0.84
NPV	0.60
Youden	0.46
Kappa	0.45

FU1 AML 4-5

Tube 1

	Mol+	Mol-
MFC+	13	1
MFC-	6	2

	Mol+	Mol-
MFC+	59.1%	4.5%
MFC-	27.3%	9.1%

Concordance 68.2%
Discordance 31.8%

Se	0.68
Sp	0.67
PPV	0.93
NPV	0.25
Youden	0.35
Kappa	0.21

Tube 2

	Mol+	Mol-
MFC+	15	1
MFC-	4	2

	Mol+	Mol-
MFC+	68.2%	4.5%
MFC-	18.2%	9.1%

Concordance 77.3%
Discordance 22.7%

Se	0.79
Sp	0.67
PPV	0.94
NPV	0.33
Youden	0.46
Kappa	0.32

Tubes 1 & 2

	Mol+	Mol-
MFC+	17	1
MFC-	2	2

	Mol+	Mol-
MFC+	77.3%	4.5%
MFC-	9.1%	9.1%

Concordance 86.4%
Discordance 13.6%

Se	0.89
Sp	0.67
PPV	0.94
NPV	0.50
Youden	0.56
Kappa	0.49

Figure S2: Display of the results for two different subgroups of patients. Top panel: Patients with NPM1 mutation only according to all time points (N=60) or FU1 only (N=29). Bottom panel: Patients with AML4 and AML5 (myelomonocytic) only according to all time points (N=47) or FU1 only (N=22). Matrices of numbers and percentages of concordance between molecular and MFC MRD detection are shown for tube 1, tube 2 and the combination of both tubes. Se: sensitivity, Sp: specificity, PPV: positive predictive value, NPV: negative predictive value. Youden and kappa tests were used and values displayed as well.

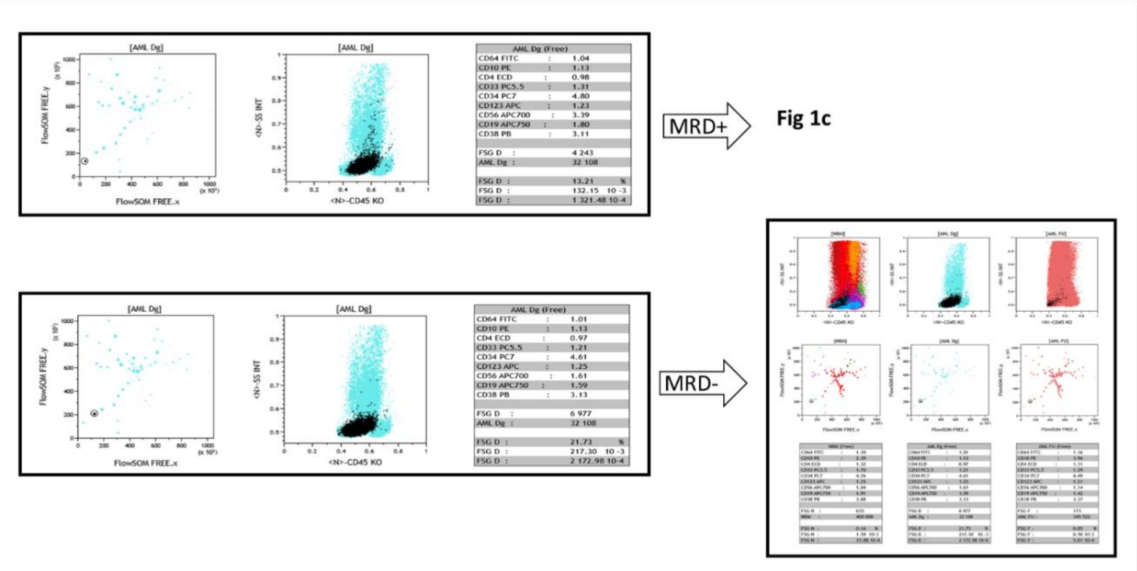

Figure S3: Unsupervised delineation of two adjacent but distinct nodes identifying subclones with different LAIP at diagnosis, respectively CD56-positive (top) and CD56-negative (bottom). The black backgating of each node on the CD45/SSC Dg histogram would not allow to distinguish these populations with classical MCF. FlowSOM identifies positive MRD for the CD56+ subclone (shown in Figure 1c) but MRD is undetectable for the CD56- subclone.
